# Application of Metabolomics in Pediatric Asthma: Prediction, Diagnosis and Personalized Treatment

**DOI:** 10.3390/metabo11040251

**Published:** 2021-04-18

**Authors:** Maria Michelle Papamichael, Charis Katsardis, Evangelia Sarandi, Spyridoula Georgaki, Eirini-Sofia Frima, Anastasia Varvarigou, Dimitris Tsoukalas

**Affiliations:** 1 Department of Dietetics, Human Nutrition & Sport, School of Allied Health, Human Services & Sport, La Trobe University, Melbourne 3086, Australia; 2 European Institute of Nutritional Medicine, 00198 Rome, Italy; dtsoukalas@einum.org; 3 Department of Experimental Physiology, Medical School, National & Kapodistrian University of Athens, 11527 Athens, Greece; katsardis@yahoo.gr; 4 Metabolomic Medicine, Health Clinic for Autoimmune and Chronic Diseases, 10674 Athens, Greece; esarandi6@hotmail.com (E.S.); sl.georgaki@gmail.com (S.G.); 5 Laboratory of Toxicology and Forensic Sciences, Medical School, University of Crete, 71003 Heraklion, Greece; 6 Department of Pediatrics, School of Medicine, University of Patras, 26500 Patras, Greece; eirinifrima@gmail.com (E.-S.F.); nata-var@med.upatras.gr (A.V.)

**Keywords:** asthma, asthma phenotypes, pediatric, metabolomics, biomarkers

## Abstract

Asthma in children remains a significant public health challenge affecting 5–20% of children in Europe and is associated with increased morbidity and societal healthcare costs. The high variation in asthma incidence among countries may be attributed to differences in genetic susceptibility and environmental factors. This respiratory disorder is described as a heterogeneous syndrome of multiple clinical manifestations (phenotypes) with varying degrees of severity and airway hyper-responsiveness, which is based on patient symptoms, lung function and response to pharmacotherapy. However, an accurate diagnosis is often difficult due to diversities in clinical presentation. Therefore, identifying early diagnostic biomarkers and improving the monitoring of airway dysfunction and inflammatory through non-invasive methods are key goals in successful pediatric asthma management. Given that asthma is caused by the interaction between genes and environmental factors, an emerging approach, metabolomics—the systematic analysis of small molecules—can provide more insight into asthma pathophysiological mechanisms, enable the identification of early biomarkers and targeted personalized therapies, thus reducing disease burden and societal cost. The purpose of this review is to present evidence on the utility of metabolomics in pediatric asthma through the analysis of intermediate metabolites of biochemical pathways that involve carbohydrates, amino acids, lipids, organic acids and nucleotides and discuss their potential application in clinical practice. Also, current challenges on the integration of metabolomics in pediatric asthma management and needed next steps are critically discussed.

## 1. Introduction

Asthma in children remains a significant public health challenge affecting 5–20% of children in Europe and is associated with increased morbidity and societal healthcare costs [1]. High variations in asthma incidence among countries might be attributed to differences in genetic susceptibility, socioeconomic disparities, health literacy, indoor and outdoor pollution (such as residing in heavily industrialized regions, traffic pollution), aeroallergen and microbial exposure, climatic conditions, use of open fires for cooking and heating [2]. Furthermore, socioeconomically disadvantaged families may not have access to medical facilities and afford to purchase medications as well as recognize that the child suffers from asthma. Asthma tends to initiate at an early age leading to more years of disability compared to other chronic diseases and subsequent negative impact on the quality of life [3]. This respiratory disorder can be described as a heterogeneous disease characterized by chronic airway inflammation that is manifested by symptoms of wheeze, dyspnea, chest tightness and cough of variable intensity and airway limitation [1]. A common denominator to all phenotypes is reduced lung function and low-grade chronic airway inflammation. In Pediatrics, diagnosis is based on reporting of patient symptoms lung function as evaluated by spirometry [decrease in peak expiratory flow (PEF) and forced expiratory volume at 1 sec (FEV_1_)] and response to pharmacotherapy [4]. However, an accurate diagnosis is often difficult due to the diversities in clinical presentation and fluctuations in PEF and FEV_1_ values. Therefore, identifying early diagnostic biomarkers and tools that will improve the monitoring of airway dysfunction and inflammation through non-invasive methods are key goals in successful pediatric asthma management. Currently, there is increasing attention on the development of methodologies that permit the close monitoring of disease-associated lung function changes [5] and the identification of novel biomarkers that will predict asthma progression on a molecular level. It is believed that asthma is driven by the interaction between genes and environmental factors, including nutrition, air pollution, and cigarette smoke, pet hair, viral infections, weather changes, dust mites, dampness, mold and high body weight [2]. In this context, asthma is suitable for the application of metabolomics in order to obtain a better understanding of the mechanisms behind asthma inception.

Metabolomics—the systematic analysis of small molecules involving carbohydrates, amino acids, lipids, organic acids and nucleotides generated from the cellular metabolic activity—is a valuable tool for biomarker discovery, predicting response to therapy and potential pathogenic pathways for a variety of complex diseases including diabetes [6], cardiovascular disease [7] and autoimmune diseases [8,9]. Metabolomic studies are typically performed on biological specimens, including blood/sera, exhaled breath and urine samples, which are amongst the most often used in pediatric research because they can be collected using simple, non-invasive techniques [10]. An additional advantage is that these biospecimens cover a large number of detectable metabolites, thus providing an accurate overview of the metabolome. The basic principle of the application of metabolomics in clinical research and practice lies in the fact that the metabolic profile in the disease state differs from the healthy. Metabolomics has been widely used for decades in pediatrics for the detection of Inborn Errors of Metabolism (IEM) during newborn screening. Detection of abnormal levels of metabolites in human biospecimens indicates dysfunction of the enzyme that is responsible for the involved biochemical reaction. In the case of IEMs, early identification of the deficient enzyme due to genetic defects can be detected by the measurement of the related metabolites. Similarly, disruptions at multiple biochemical pathways by the reduced activity of related enzymes caused by non-genetic factors are being increasingly associated with several chronic diseases [11]. The development of state-of-the-art metabolomics technologies has facilitated the capturing of small-scale metabolite fluctuations, which can be used as potential biomarkers for disease prediction and diagnosis.

Emerging analytic techniques and improved statistical analysis are now available for quantifying novel biomarkers, thus rendering the metabolomics approach to be a new and potentially exciting science in pediatric respiratory disease [10]. Moreover, because of the high sensitivity of metabolomics, subtle changes in biological pathways can be detected to disentangle the mechanisms involved in various physiological conditions and in disease [10].

**Figure 1:**
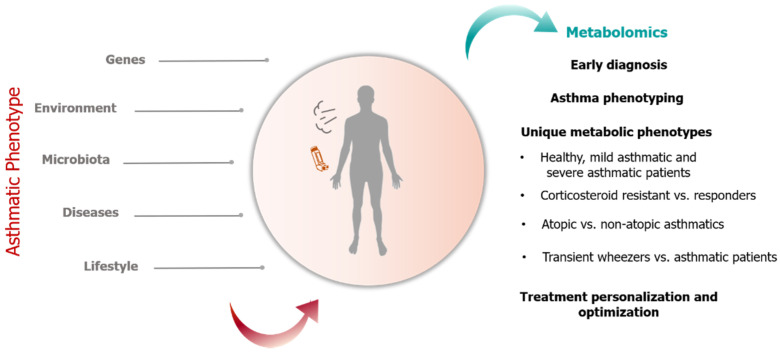
Genetic and non-genetic factors modulate the asthmatic phenotype which can be evaluated through metabolomics. Application of metabolomics in pediatric asthma include early diagnosis, asthma phenotyping through identification of unique metabolic fingerprints and treatment personalization and optimization.

Metabolic profiling is appropriate for the study of complex diseases with a genetic and environmental etiology such as asthma because it has the potential to capture the history of the cellular response to past exposures [12]. Furthermore, metabolite fluctuations reflect genetic and environmental interactions, generating a metabolic fingerprint that could illuminate the role of molecular mediators in asthma pathobiology, which at present is poorly understood (Figure 1).

The purpose of this review is to provide evidence on the utility of metabolomics in pediatric asthma and discuss their potential application in clinical practice. Current challenges on the integration of metabolomics in pediatric asthma management and needed next steps are also addressed.

### 1.1. Strategies in Metabolomics Research

Metabolomic studies can be categorized into two analytical approaches, untargeted versus targeted. These strategies differ in study objective (discovery or hypothesis testing), quantification level, sample preparation, accuracy and precision of the obtained results, along with the number of metabolites identified [13]. Untargeted metabolomics measures a broad range of metabolites in a biological sample for a specific physiological state under given environmental conditions [14]. An advantage of this method is that it allows for the investigation of the complex interaction between metabolites from multiple pathways prior to hypothesis formation. Although this approach allows for novel metabolite discovery, technically, it is impossible to recover all metabolites [14]. The main limitation of untargeted metabolomics is the generation of a large amount of data that is difficult to analyze. Moreover, untargeted approaches require overcoming several experimental difficulties, including the identification and characterization of previously unknown metabolites, which partly depends on the efficiency of the analytical method employed and on analyzing the entire range of metabolites avoiding the biased selection of high-abundance molecules [14]. Nonetheless, several studies on pediatric asthma have employed untargeted metabolomics as a tool to discriminate different phenotypes and cases based on disease severity [15,16].

Conversely, targeted metabolomics is the measurement or quantification of pre-defined known groups of metabolites of a related chemical structure and biologic activity. This method has several advantages over untargeted metabolomics, including higher sensitivity and selectivity. Metabolites are analyzed based on a priori knowledge of specific metabolites, enzymes and kinetics, end products and biochemical pathways. Furthermore, in targeted metabolomics, sample preparation method modifications decrease the number of high-abundance molecules and techniques such as triple quadruple mass spectrometry provide sensitive quantification of molecules that are very low in a sample hampering their detection. Most importantly, using pre-defined metabolites, novel associations between molecules can be used to explain observations in the context of specific diseased states. With respect to asthma research, targeted metabolomics using exhaled breath successfully detected a distinct profile of volatile organic compounds in pre-school children with recurrent wheeze at age 6 years as compared to healthy children with no wheezing [17].

The main methodologies employed for metabolite recovery and identification in a biospecimen are nuclear magnetic resonance spectroscopy (NMR) and mass spectrometry (MS) coupled with chromatographic separation (liquid (LC) or gas chromatography (GC)) [13]. NMR strategies provide the potential of acquiring unanticipated results regarding the changes of metabolites and offer an overall view of the metabolome under specific conditions. ^1^H-NMR spectroscopy characterizes proton-containing low-molecular-mass compounds in a biological sample, which are represented in a spectrum [13]. A drawback is that NMR is less sensitive than MS, and the NMR spectra can produce overlapping signals among metabolites rendering quantification difficult [13]. Alternatively, it has high reproducibility, being non-selective in detection for specific metabolites a broad range of molecules can be identified and measured simultaneously, requires minimal sample preparation, short measurement time, as well as small sample volumes, can be analyzed [13]. Moreover, it is able to classify phenotypes.

In contrast, MS separates molecules in a biological sample and their fragments on the basis of their mass-to-charge ratio, which is presented as a spectrum [13]. This technique has higher sensitivity, reproducibility and selectivity than NMR [13]. Specifically, LC-MS and GC-MS platforms require minimal sampling techniques compared to the wide collection of metabolic fluctuations that can be assessed. LC-MS is also robust, thus providing the optimal toolset. The main downsides include the increased costs for a fully equipped laboratory and the reduced levels of fragmentation in LC-derived ionization, utilized for the determination of molecular and structural data of the obtained metabolites [13].

### 1.2. Application of Metabolomics in Pediatric Asthma Research

Metabolomics is an appealing application to monitor environment–gene interactions since measured metabolites reflect metabolic alterations and dysfunction in various organs and cells that are associated with the development of diseased states. With respect to asthma research, metabolomics offers the potential to identify biomarkers for host susceptibility, assess response to environmental risk factors, and monitor the development of wheeze and asthma, including highlighting the underlying mechanistic pathways. Studying early life respiratory morbidity in infants is challenging. In infants, in vivo experiments with environmental exposures (for example, viral infection) are not feasible due to ethical restrictions [18]. Moreover, investigations in animal models [18] or adults cannot be extrapolated to infant and child populations because of differences in immune systems, lung development and diversity in response to environmental exposures based on age. On this note, metabolomics offers an attractive approach throughout the lifecycle in asthma research.

## 2. Results

Of the 20 relevant publications identified on the topic of interest, seven were cohorts [19,20,21,22,23,24,25], eight cross-sectional [15,16,26,27,28,29,30,31], two prospective [32,33], one case-study [34], longitudinal [35] and experimental [36]. Three studies were conducted in the USA [15,25,31], two in Taiwan [26,35], five in Italy [16,21,30,32,34], four in the Netherlands [19,22,23,33], one in Canada [27], China [24], UK [36], Greece [28], Costa Rica [29] and Denmark [20]. Eight studies investigated differences in metabolic profiles between asthma versus healthy controls [19,20,24,26,27,33,35,36], three studies were on transient wheezers versus early onset asthma ([21,22,23], three focused on bronchiolitis [25,32,34], while six studies were on asthma phenotypes [mild (*n* = 2) [28,29], severe (*n* = 1) [30], corticosteroid resistant (*n* = 2) [15,31] and atopic asthma (*n* = 1)] [16]. Two studies focused on neonates [19,20], four were on infants [25,32,34,35] and preschoolers [21,22,23,26], whereas ten studies included school children [15,16,24,27,28,29,30,31,36]. Ages ranged from 0–17 years. Regarding biological specimens obtained from subjects, one study examined amniotic fluid, [19] three blood [15,26,29], twelve urine [16,20,21,24,25,26,27,28,31,32,34,35], one exhaled breath condensate (EBC) [30] and four volatile organic compounds (VOCs) [22,23,33,36]. Metabolites were detected employing Nuclear Magnetic Resonance (NMR) (*n* = 5) [25,26,27,34,35] and Chromatography-Mass Spectrometry [GC-MS (*n* = 6) [22,23,24,28,33,36] and LC-MS (*n* = 9) [15,16,19,20,21,29,30,31,32] metabolomic profiling techniques, of which 14/20 used a targeted approach. Details of study characteristics and significant metabolites identified are summarized in Table 1 and with respect to asthma phenotyping in Table 2. 

## 3. Discussion

**Figure 2:**
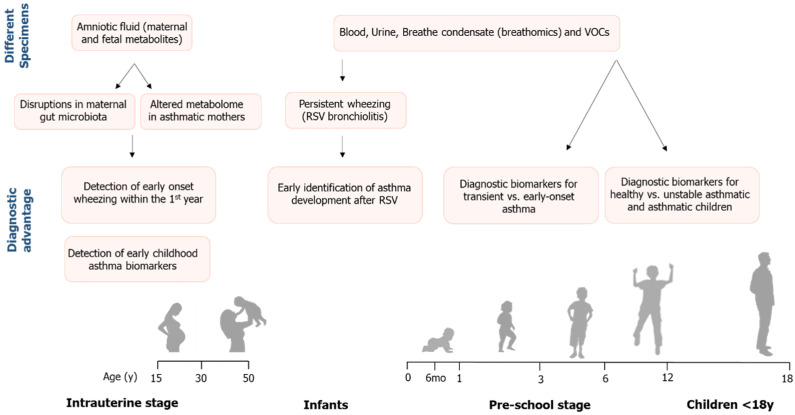
Summarizing diagram showing the application of metabolomics in patients with asthma at the intrauterine, pre-school stage and in children. Depending on the stage and the biospecimen type, metabolites can be potent predictive biomarkers or tools for asthma phenotyping.

Asthma is a complex chronic respiratory disease with genetic and environmental etiology, and due to the limited understanding of its pathophysiology, novel tools are warranted for its detection and treatment. The unique advantage of metabolic profiling is that it provides a snapshot following exposure to an environmental stimulus, impact on the immune response and airway function. Thus, it leads to a better understanding of the underlying pathogenic mechanisms that contribute to asthma inception and potential targets for disease prevention. This review presented metabolomics, an emerging field with vast application in health and disease, and its utility in pediatric asthma research (Figure 2). 

Intriguingly, the findings of these studies suggest the presence of metabolic perturbations in early life even before birth, heralding the onset of asthma symptoms. We demonstrated that metabolic profiling of pediatric asthma patients from intrauterine life to childhood successfully identified putative biomarkers that distinguished between wheezing, asthma and healthy children as well as had high discriminative ability in characterizing asthma phenotypes. A plethora of metabolites was associated with asthma status, severity, exacerbations, and phenotype discrimination, suggesting that these metabolites might be key players in asthma onset as well as development. Although discordance existed in metabolites among studies and metabolic profiles varied upon the biospecimen assessed, there were consistencies in mechanistic pathways in all age groups. Overall, studies described suggest that asthma caused alterations in metabolic pathways related to carbohydrate, protein, purine, and lipid metabolism, steroid and glutathione synthesis, as well as one-carbon folate metabolism. Furthermore, some metabolites were products of gut microbiota metabolism and others associated with epigenetic dysregulation, thus suggesting that changes in DNA methylation might contribute to asthma induction. Potentially, fluctuations in annotated pathways are attributed to cardinal features of asthma pathogenesis that include hypoxia, oxidative stress, immune and inflammatory processes. 

### 3.1. Hypoxia and Energy Deficits

#### 3.1.1. Citric Acid Cycle

In the milieu of hypoxia, edema, bronchoconstriction, airway hyperresponsiveness coupled with the increased mechanical work of breathing are characteristics of asthma that lead to increased energy demands. In response to these events, cellular energy deficits were evident by increases in intermediates of the citric acid cycle (citrate, isocitrate, aconitic, succinate, oxo-glutarate, pyruvate, malic) [21,25,26,27,28,32], protein metabolism (creatine, branched-chain amino acids (BCAA), E-2-methylglutconic acid, 3-hydroxy-*N*6, *N*6, *N*6-trimethyl-l-lysine, *N*2-acetyl-ornithine, *N*-acetylputrescine) [19,21,24,26,32] and fatty acid oxidation [20,21,24,27,29]. Collectively, representing the increased rate of glycolysis, gluconeogenesis and β-oxidation to replenish energy needs of inflamed airways [37] under hypoxic conditions [38]. Alternatively, these fluctuations might reflect the inability of the damaged lung to meet the energy demands of activated inflammatory cells in airways [39]. In murine models, it was demonstrated that asthma affected energy production and metabolism of carbohydrates, amino acids, lipids and sterols in the lungs [39]. Disruptions in the citric acid cycle and pyruvate metabolism have been reported in adult studies investigating the application of metabolomics in asthma [37,40].

Citrate plays a pivotal role in energy production, synthesis of proinflammatory molecule prostaglandin E2 (PGE2) and for oxaloacetate to generate NADPH via acetyl-CoA [41]. Notably, studies reported increased succinate concentrations in asthma groups underlining the high turnover rate of the citric acid cycle and abundance of intermediates to supply for the increased work of breathing during exacerbations and oxygen debt [27,37,40]. In addition, citrate is a substrate for the production of itaconate via cis-aconitate, which acts as a modulator of inflammation by down regulating inflammatory mediators [42]. With respect to RSV-induced wheeze [25], low levels of citrate and increased cis-aconitate could represent citrate depletion and consequently reduction in the production of pro- and anti-inflammatory mediators required to counteract viral replication. 

In cellular respiration, each step of the citric acid cycle requires enzymes and cofactors or vitamins for the production of energy in the form of Adenosine Triphosphate (ATP). Vitamins B5 and pantothenic acid are important in carbohydrate and fatty acid metabolism. It is required for the synthesis of coenzyme A (CoA), which is an important intermediate of the citric acid cycle, and acyl carrier protein of fatty acid synthase [43]. Increased synthesis of pantothenate and coenzyme A [24] would indicate increased energy needs in asthmatic patients, possibly attributed to the increased work of respiration. In addition, levels of vitamin B1 and thiamine discriminated against the mild-moderate asthma phenotype [29]. Thiamine serves as a cofactor for enzymes involved in the metabolism of carbohydrates promoting energy production and the synthesis of fatty acids, nucleic acids and amino acids.

In the setting of poor tissue oxygenation, elevated lactate levels [26,27,28] reflect anaerobic glycolysis. Moreover, high lactate and malate are products of altered energy metabolism produced during abnormal lung respiration under conditions of inflammation and hypoxia [39]. Correlation analysis has revealed strong positive correlations between lactate, malate and the immune response (macrophages and eosinophils) [39]. Interestingly, we found a strong correlation between urinary malate concentrations and deterioration of asthma control (as evaluated by a questionnaire) in mild-asthmatic children [28]. The oxidation of lactate to pyruvate and ultimate conversion to glucose via the Cori cycle [44] is enhanced by inosine, a breakdown product of adenosine. Inosine levels were upregulated in asthma [37]. Inosine is known to enhance the activity of coenzyme A and pyruvate oxidase, thus facilitating cellular respiration under hypoxic conditions [37]. Malate is an intermediate of the citric acid cycle and participates in the malate-aspartate shuttle, which is important in transferring electrons produced by glycolysis into the mitochondrial inner membrane to generate ATP [39]. Therefore, elevated malate might indicate mitochondrial dysfunction due to hypoxia, increased reactive oxygen species in airways, and energy depletion attributed to the increased work of breathing [45]. Additionally, decrements in fumarate and succinate would correspond with increased synthesis of malate during asthma exacerbations. Regarding lung function, Kelly et al. reported associations between intermediates of the citric acid cycle and FEV_1_/FVC in children of the mild-moderate asthma phenotype [29]. In muscles, when energy is depleted or, during anaerobic exercise, lactate is elevated, alanine concentrations increase due to gluconeogenesis along with increased phosphorylation of creatine to phosphocreatine [44]. It is worth noting that high levels of creatine kinase, the enzyme which converts creatine to phosphocreatine via phosphorylation [46], and the presence of myoglobin in urine were documented in adult patients suffering from severe acute asthma [47]. A possible explanation might be that during severe asthma attacks, rapidly repeated contractions of the diaphragm and accessory muscles of respiration are comparable to strenuous exercise [48].

Furthermore, depleted energy stores were mirrored by increased levels of 1-methyl nicotinamide, pantothenate (a precursor of CoA) and CoA synthesis, which are essential for the metabolism of carbohydrates, protein and lipids, important substrates for energy production. Interestingly, Kelly et al. found that pantothenate and CoA biosynthesis were enriched and associated with FEV_1_/FVC in children of the mild-moderate asthma phenotype [29], possibly signifying energy depletion with bronchial obstruction and increased energy requirements of activated inflammatory cells [39,49].

#### 3.1.2. Nicotinamide

Metabolites involved in the metabolism of nicotinate and nicotinamide were markedly decreased in infants with RSV-induced bronchiolitis and school children compared to healthy controls [25,26,27,35]. High nicotinamide levels were reported in plasma of adult asthmatics compared with healthy controls [50]. 1-Methylnicotinamide, a derivative of nicotinamide, is a precursor in the synthesis of Nicotinamide adenine dinucleotide (NAD^+^) and NAD phosphate (NADP^+^), which are key players in energy production via the electron transport chain and regulation of cellular redox [51]. A prophylactic effect of 1-methylnicotinamide on asthma exacerbations has been reported, most likely due to the anti-inflammatory effects of mannose-binding lectin levels [52]. Apart from anti-inflammatory properties, 1-methylnicotinamide acts as a potential scavenger of reactive oxygen species resulting in the inhibition of lipid peroxidation [53] as well as reduces adherence of pro-inflammatory cells and molecules to the surface of the vascular endothelium [51]. In animal models, 1-methylnicotinamide was found to prevent asthma exacerbations in mice [52]. Thus, low concentrations of 1-methyl nicotinamide in asthmatic infants and children could signify increased consumption of NAD+ because of increased energy needs to support respiratory muscle work along with increased airway inflammation. With respect to mitochondrial dysfunction, glycolate, carnitine, and O-acetyl-carnitine [27] participate by transporting fatty acids into mitochondria for oxidative phosphorylation in the production of energy in the form of ATP [54,55].

Notably, increased acetone and decreased 1-methylnicotinamide levels were observed in infants with RSV compared to the healthy group [25]. Under unfavorable conditions, when glucose and oxaloacetate levels are low, acetone is produced [44] and used as an alternative substrate to fuel energy production pathways and maintain the normal function of vital organs [56]. It is possible that, in RSV infants, levels of acetone increase while 1-methyl nicotinamide levels decrease in response to the high inflammation attributed to stress from the viral infection, maintaining the normal energy supply [25]. 

### 3.2. Protein Synthesis/Degradation

Abnormalities in amino acid metabolism overlapped in studies reviewed throughout childhood and paralleled with adult asthma patients [37,40]. One could speculate that under hypoxic conditions, increased respiratory muscle work due to dyspnea, coupled with an energy deficit, would result in protein degradation as an auxiliary route for gluconeogenesis and energy production [44]. Creatine was identified in the plasma of mild-moderate asthma patients [29]. This amino acid is involved in muscular protein turnover and energy supply to muscles, including airway smooth muscles. Therefore, increases in pulmonary levels of creatinine, a downstream metabolite of creatine, suggest the promotion of energy metabolism via the urea cycle [39]. On the contrary, studies have reported that creatinine amplified lung inflammation, hyperresponsiveness and airway remodeling through T-helper-2-type (Th2) activation and increased proinflammatory cytokines [57,58]. Guanidoacetic acid, a precursor of creatine is synthesized from arginine and glycine [59]. Pertaining to AHR, disturbances were noted in d-glutamine/glutamate metabolism of mild-moderate asthmatic children [29], reflecting airway obstruction [60]. Reduced levels of glutamine have been reported in adult asthma, signifying alterations in amino acid metabolism, probably due to the high rate of gluconeogenesis to support respiratory muscle work and airway epithelial repair [37,40]. Intracellularly, glutamine is concentrated in skeletal muscle and utilized for a variety of physiological processes ranging from citric acid intermediates, NADPH synthesis via glutaminolysis, nucleotide synthesis and maintenance of redox homeostasis mediated by glutathione synthesis, as well as in fatty acid synthesis [61]. Moreover, glutamine is used as an energy source by immune cells, namely lymphocytes, neutrophils and macrophages [61]. Therefore, decreases in glutamine levels might reflect impaired immune cell function in asthma.

Surprisingly, alterations in protein metabolism in urinary profiles of neonates [34] and infants [25] affected with RSV-induced bronchiolitis were also observed and have been previously reported [62]. In fact, alanine, tyrosine, and 4-deoxythreonic acid were associated with recurrent wheezing in the first year of life in infants with RSV bronchiolitis [25], which could reflect inflammation present in bronchioles. Alanine was also detected in children of the mild-moderate asthma phenotype [29]. In adult studies, alanine discriminated between healthy and severe asthma [50], with decreased levels reflecting abnormal amino acid metabolism [63]. From another point of view, elevated levels of 4-deoxythreonic acid were recovered from urinary organic acid profiles of patients with juvenile-onset Type 1 diabetes mellitus [64] and insulin resistance (as measured by HOMA-IR index) has been observed in obese asthmatic children [65].

In addition, changes in creatine metabolism were detected in preterm neonates [34]. Regarding creatinine recovered in urine samples of neonates, this metabolite is a constituent of muscle tissue that is excreted by the kidneys [66]. Prenatal and neonatal events, together with genetic factors, have been known to influence renal development and function in neonates [67]. Nephrogenesis is dependent on gestational age and intrauterine environment [68]. In this context, changes in creatinine levels of neonates might reflect nephrogenesis [67]. Interestingly lower glomerular filtration rate and disturbed tubular function were found in school children born prematurely [69]. Notably, apart from respiratory distress, the presence of amino acid metabolites in neonatal urine could be the outcome of fetal maturation processes.

In pre-school children, Carraro et al. reported high levels of intermediates related to tryptophan metabolism (5-hydroxy-l-tryptophan, indole-3-acetamide, and 3-indoleacetic-acid, indole, glutaric acid, 5-hydroxy-1-tryptophan, indole-3-acetamide, kynurenine and 3-indoleacetic) in early-onset asthma, while indoleacetic a breakdown product of tryptophan and tyrosine metabolism was associated with transient wheezing [21]. Prior studies have documented upregulated synthesis of tryptophan in uncontrolled asthma than in healthy controls [15,26,70,71]. Tryptophan is an important substrate for the synthesis of coenzymes NAD and NADP as well as for serotonin, dopamine, norepinephrine and melatonin production, which regulates circadian rhythms and influences the immune system [72]. Asthma research shows that tryptophan participates in inflammation, oxidative stress [70] and is an important mediator in the immune response [73]. Tryptophan metabolism was markedly altered in patients with allergic asthma compared to controls, which was a favorable factor against rhinovirus infection. Specifically, higher levels of tryptophan and its downstream metabolites were positively associated with eosinophilia and asthma control scores after experimental infection with rhinovirus [74]. Kynurenine is formed from tryptophan degradation by the enzyme indoleamine 2, 3, dioxygenase and is the precursor for the synthesis of NAD [75]. Dysregulation or overactivation of the kynurenine pathway can lead to activation of the immune response [75]. High exhaled nitric oxide (eNO) levels are features of atopic asthma, and suppression of tryptophan-degrading enzyme indoleamine 2, 3-dioxygenase-1 (IDO-1) by NO could explain high tryptophan levels in childhood asthma [76]. This is important because tryptophan and IDO-1 are strongly involved in immunomodulation [76]. In a study of 205 children (4 months to 17 years), tryptophan and kynurenine levels were higher, and Ig E and IDO activity lower in those with asthma and allergic rhinitis [77]. From a different perspective, amino acids are susceptible to oxidative damage by reactive oxygen species. Therefore, modification of amino acids could consequently lead to an increase of urinary oxidation products in asthma patients [78]. As for the negative correlation between glycolic acid and spirometry measure PEF [28], this metabolite is a mediator in the tyrosine pathway [79]. Tyrosine is a precursor of catecholamines that are released under conditions of stress (flight-to-fight response) and are involved in the regulation of the immune system [80]. Regarding hydroxyindoleacetate [28], this molecule is the end product of serotonin metabolism. Serotonin is generated from the breakdown of tryptophan and plays a central role in signaling the immune response by modulating chemotaxis, leukocyte activation, proliferation, cytokine secretion, anergy, and apoptosis [81].

Comparable to adult asthma [37], phenylalanine, another essential amino acid, was detected in asthmatic children [27] and in those of the mild-moderate asthma phenotype [29], while 3,4-dihydro-l-phenylalanine differentiated corticosteroid resistant severe asthma phenotypes [31]. Phenylalanine is critical in the production of tyrosine and catecholamines, including dopamine [44,79], and urinary 3-phenylpropionate [26] is an end product of phenylalanine and tyrosine degradation [82]. During conditions of increased stress, catecholamine release was related to bronchoconstriction [79]. Potentially, decreasing levels of 3,4-dihydro-l-phenylalanine in response to asthma therapy could potentially predict clinical responsiveness to inhaled corticosteroids (ICS) and deserves future investigation. In the event of an asthma attack, eosinophil recruitment and production of NO-derived oxidants are stimulated [83]. Activated eosinophils degranulate, releasing eosinophil peroxidase, which converts hydrogen peroxide to the reactive brominating oxidant, hypobromous acid that modifies protein tyrosine residues forming 3-bromotyrosine [84]. Urinary bromotyrosine can be used as a molecular fingerprint for eosinophil activation [84], a predictor of asthma, asthma control and future exacerbations in children [71]. It has been reported that urinary bromotyrosine levels corresponded with asthma control scores in pediatric patients with asthma [71]. In particular, high bromotyrosine levels were associated with 5.0-fold odds of inadequately controlled asthma, increased symptoms, activity limitation, and medication use, as well as 4-fold odds of having an exacerbation in the next six weeks [71]. Therefore, high urine tyrosine levels in patients would represent a state of inflammation and oxidative stress associated with asthma pathogenesis. 

With respect to metabolites involved in histamine biosynthesis, urinary histidine, imidazole, methyl-imidazole acetic acid, 1-methylhistamine were related to asthma and the atopic asthma phenotype [16,26,27]. Approximately 70–80% of histamine metabolized is excreted in urine as methyl-imidazole acetic acid [85]. After exposure to an allergen, histamine is released from mast cells and evokes bronchoconstriction in the allergic response through airway smooth muscle contraction, increased secretion from airway submucosal glands as well as activation of dendritic cells, B cells, Th1 and Th2 lymphocytes through H1 and H2 receptors on the cell surface [86]. Prior studies have documented high 1-methylhistamine serum levels in pediatric asthmatic patients after asthma exacerbations [26,40]. Paradoxically, Mattarucchi et al. reported reduced levels of methyl-imidazole in atopic asthma. This might be explained by the H4 receptor that is expressed by inflammatory cells. Low histamine levels appear to induce recruitment of dendritic cells, eosinophils and mast cells facilitated by the H4 receptors [87]. Therefore, low concentrations of urinary methyl-imidazole acetic acid in atopic asthma would be a marker of alterations in histamine metabolism. As for the third metabolite reported by Mattarucchi found in atopic asthma patients, Isoleucyl-Proline (Ile-Pro), it has been speculated that it is related to prolidase activity which participates in collagen degradation during airway remodeling [88]. The accumulation of collagen in the airways causes a reduction in prolidase activity [88] which was evident by the decrease in Ile-Pro fragments recovered in urinary samples of children.

Tao et al. reported elevated levels of branched-chain amino acids BCAA leucine, valine and isoleucine in uncontrolled and well-controlled asthma, while serine and threonine were upregulated in uncontrolled asthma [24,27]. BCAA are essential amino acids necessary for protein synthesis, as key nitrogen donors involved in the intercellular shuttling of nitrogen and as an anabolic molecule for nutrient-signaling that stimulates protein synthesis in selected tissues. According to previous studies, BCAA administration was associated with improved glucose metabolism [89] and glucose uptake by skeletal muscles [90], had a positive effect on the antioxidant cellular mechanisms, thus reducing the oxidative stress effects [91] and improved the immune system response of compromised patients [92]. In vitro and animal studies indicate that BCAA are important for the innate immune response as well as efficient immune function [93]. With respect to asthma, increased BCAA could simply indicate allergy-related outcomes [26].

Concerning low levels of arginine, l-ornithine, *N*-acetyl-ornithine and l-citrulline in children with early-onset asthma than in healthy controls [21], this would also represent reduced urea and nitrogen metabolism, which coincides with previous pediatric and adult asthma studies [37,40,94,95]. Plausibly, airway damage due to frequent exacerbations would increase protein needs for tissue repair.

On the contrary, significantly higher taurine levels were detected in asthmatic children [27] and adults [50]. Taurine is classified as a non-essential amino acid that is not required for protein synthesis [96]. It is abundant in the brain, muscle tissue and organs of the body and is necessary for the normal functioning of the central nervous system [96]. Taurine has a variety of properties ranging from osmolyte involved in cell volume regulation, as a precursor for bile salt synthesis, modulation of intracellular calcium concentration, cytoprotection and acts as an anti-oxidant [96]. Although the role of taurine in asthma is unclear, it can be speculated that taurine might serve as a biomarker for inflammation and increased oxidative stress in airways. Interestingly, plasma taurine levels were positively correlated with arachidonate (omega-6 fatty acid) in adult patients suggesting a role in inflammation [50]. 

### 3.3. One-Carbon Folate Cycle

Methylating agents (serine, glycine, betaine, S-adenosylhomocysteine (SAH) and methionine) were replicated in pediatric, and infant studies reviewed [15,24,27,32,34] as well as related to FEV_1_/FVC in the mild-moderate asthma phenotype [29]. These crucial amino acids play a fundamental role in the one-carbon folate and methionine cycle in the generation of S-adenosylmethionine (SAM), a universal donor for methylation reactions including histone and DNA/RNA methylation [97]. Serine is a non-essential amino acid that is a precursor for many biosynthetic and signaling pathways, including the folate cycle that supports nucleotide and protein synthesis, methylation reactions, membrane lipid synthesis, antioxidant defense and indirectly maintenance of redox status (NADPH/NADP^+^) [97]. During acute stress, serine needs are increased. The folate cycle is initiated by one-carbon group transfer from serine to tetrahydrofolate, which after a series of reactions results in 5-methyltetrahydrofolate, the active form of folate and prime methyl donor for the methylation of homocysteine to methionine [97]. Then, methionine is converted to SAM [97]. Glycine participates in the folate cycle as glycine N-methyltransferase that converts SAM to SAH, after which homocysteine is formed and converted to methionine [97]. Betaine is required as a methylating agent for the latter reaction [97]. Both betaine and threonine can be used by cells to form glycine via a series of demethylation reactions. The final end products of SAH hydrolysis are adenine and homocysteine, whereas homoserine metabolized from methionine serves as an intermediate for threonine synthesis [98]. This brings to mind that threonine differentiated corticosteroid-resistant pediatric asthma patients versus mild asthmatics [15], which could be linked to fluctuations in the folate cycle.

Incidentally, diets high in methionine and low in folate and cobalamin, known as vitamin B12 (the co-factor for converting homocysteine to methionine) cause high SAH concentrations and, consequently, low methylation rates [99]. Hypoxia, a characteristic of asthma, is also known to influence demethylation [99]. High levels of SAH were identified in the amniotic fluid of neonates that continued to develop wheeze at 1 year [19]. It is possible that during intrauterine life, maternal diets low in folate, choline, vitamin B12 and betaine could trigger abnormal DNA methylation priming, a background for early onset of wheezing [100]. Therefore, assesing the levels of these nutrients might reflect DNA methylation and offer potential preventive and therapeutic targets in pathological conditions, including childhood asthma.

Another important function of serine is as a substrate for the production of glycine and cysteine, which together with glutamate are precursors for glutathione, a potent antioxidant [101]. So, in uncontrolled asthma, high serine levels could be a marker for increased oxidative stress associated with asthma, depletion of energy stores attributed to the increased work of breathing and dyspnea, including epigenetic alterations that favor asthma progression. 

In the domain of epigenetics, formate could participate in epigenetic regulation facilitated by DNA methylation [40,102,103]. It has been documented that formate, choline, methionine, O-phosphocholine and methanol acted as methyl donors [104], while increased arginine methylation participated in asthma by regulating cytokine expression [94,105]. Decreased serum arginine levels were observed in adult asthma patients [40]. Prior studies suggest that DNA hypermethylation skewed immune responses towards a Th2 cell-mediated pro-inflammatory response and consequently enhanced airway inflammation [106]. Therefore, hypermethylation may represent a novel epigenetic mechanism in asthma pathogenesis.

### 3.4. Purine Metabolism

Regarding abnormalities in purine metabolism [24,25], this could be related to increased inflammation and oxidative stress associated with asthma. Uric acid and its metabolites, hypoxanthine, xanthosine, inosine and adenosine, were detected as potential urinary biomarkers in asthma patients. Notably, low concentrations of uric acid were found in uncontrolled asthmatics, suggesting inhibition of xanthine oxidase synthesis or increased consumption [107]. This is consistent with other studies reporting decreased serum uric acid concentrations in asthma patients based on disease severity [108,109]. High uric acid concentrations were correlated to severe asthma exacerbations in adolescent and adult patients than in healthy controls and inversely with lung function [108,109]. The role of uric acid in asthma remains controversial, with some studies demonstrating that high levels of uric acid-induced inflammation and oxidative stress via activation of Th2 cell-mediated immune response [110] as well as related to asthma-induced hypoxia due to degradation of adenosine [109].

Taking into consideration asthma phenotypes, adenosine, an endogenous signaling nucleoside, is a constituent of all human cells, and adenosine receptors are present in respiratory cells and on inflammatory cells [111], thus suggesting a putative role in airway inflammation. High adenosine levels have been found in bronchoalveolar lavage fluid (BALF) and exhaled breathe condensate of adult asthma patients [112,113]. Under conditions of hypoxia or high energy demands, intracellular adenosine monophosphate (AMP) is metabolized to adenosine [111]. In addition, adenosine release is also influenced by NO [113]. In children with severe asthma, NO in-breath is considerably higher than in non-severe asthmatics and healthy children, connoting a state of inflammation [30]. Substantial evidence suggests that adenosine promotes mast cell activation, enhanced histamine release, which contributes towards airway obstruction, hyperresponsiveness followed by asthma symptomology [111]. Furthermore, many other cells involved in airway inflammation are regulated by adenosine, such as neutrophils, eosinophils, lymphocytes and macrophages [111].

Inosine is a key metabolite of purine metabolism that is produced from adenosine by the catabolic enzyme adenosine deaminase (ADA), which is also linked to the inflammatory response in animal models of asthma [107]. In the occurrence of inflammation, inflammatory cells induced adenosine triphosphate (ATP) degradation resulting in increased levels of adenosine and subsequent increase in plasma inosine. Thus, high levels of inosine may be indicative of the inflammatory response of asthma [107]. Contrastingly, adenine is a precursor of adenosine, a purine molecule important in cellular respiration [44], which was detected in urine samples of asthmatic children [21,27]. It has been suggested that urinary adenine might underline abnormalities in DNA methylation reactions skewing immune responses favoring asthma progression. On the other hand, adenosine exhibits anti-inflammatory and anti-oxidant properties along with pro-inflammatory for mast cell stimulation [114]. In the case of cellular damage, adenosine concentrations increased [114]. As for urinary 6-methyladenosine noted in early-onset asthma [21], this molecule is a product of tRNA degradation [115]. 

Regarding hypoxanthine discovered in infants with bronchiolitis [25]. This metabolite is a purine derivative of adenosine metabolism and in the formation of nucleic acids [116]. In humans, hypoxanthine is involved in purine nucleoside phosphorylase deficiency, a metabolic disorder where fluid accumulation in the space between the lungs and the chest wall (pleural effusion) causes dyspnea [116]. Hence it is possible that hypoxanthine represents purine nucleoside phosphorylase deficiency in asthmatics and warrants further investigation.

Comparatively, allantoin [35], another purine metabolite, attenuated lung inflammation (↓eosinophils, lymphocytes and inflammatory cell influx), IgE and Th2 cytokines (IL-4, IL-5) in BALF of ovalbumin-induced lung inflammation in murine models of asthma [117]. Intriguingly, the effectiveness of allantoin was similar to montelukast, a leukotriene receptor antagonist resulting in decreased inflammation and smooth muscle relaxation. Notably, allantoin and urea are products of uric acid catabolism [118] and elevated levels of allantoin and uric acids along with decreased urea were indicative of perturbations in gut microbiota [118].

### 3.5. Lipid Metabolism and Inflammation

It is well-recognized that lipids participate in energy production and are key drivers of inflammation in asthma [119]. When comparing the non-severe asthma group to severe asthma, Carraro et al. measured metabolites in EBC related to inflammation, 20-hydroxy-PGF2a, thromboxane B2, and 6-keto-prostaglandin F1a [30]. The occurrence of inflammatory mediators in EBC is a common phenomenon in pediatric [120] and adult asthma [121,122]. A high omega-6 to omega-3 fatty acid ratio has been suggested to be pro-inflammatory and pro-allergic. [123]. Omega-6 polyunsaturated fatty acid, arachidonic acid gives rise to pro-inflammatory eicosanoids prostaglandins (PG2), leukotrienes (LT4) and thromboxanes (TX2) [124]. Thromboxane B2 is a metabolite of thromboxane A2_,_ which is a potent bronchoconstrictor and causes airway smooth muscle cell hyperplasia [124]. Prostaglandin PGD2 also induces bronchoconstriction, vasodilation and airway hyperresponsiveness [124]. Increased levels of TXB2 have been reported in adult patients with asthma [125]. Arachidonate was 1.5 times higher in plasma of adult asthma patients [50]. 

With respect to stearic acid [24], in vitro studies have shown that it exerts protective roles on cortical neurons by enhancing the antioxidant cellular mechanisms [126]. From a dietary aspect, high saturated fat intake as represented by burger and fast food consumption ≥ 3 times per week was associated with a 42% increase in asthma prevalence and 27% increase in severe asthma in school children (8–12 years) [127,128]. Fast food is rich in saturated fats, Trans fats and omega-6 fatty acids, which give rise to pro-inflammatory eicosanoids, Th2 cytokine production and increased airway activity resulting in asthma induction [129]. Reinke et al. reported high concentrations of saturated acids myristoic and palmitic acid and in mild, moderate and severe adult asthma patients than in healthy controls [121].

Contrastingly, omega-3 α-linolenic acid derived fatty acids, Eicosapentaenoic acid (EPA) and Docosahexanoic acid (DHA) found in fatty fish compete with arachidonic acid in cell membranes and activate the production of anti-inflammatory eicosanoids (3-series prostaglandins, 5-series leukotrienes) and bioactive molecules (protectins, maresins and resolvins) which promote resolution of inflammation and reduction in airway responsiveness [119]. We demonstrated in a recent meta-analysis that a high intake of omega-3 fatty acids mediated by fatty fish consumption reduced asthma and wheeze in young children < 5 years old [130]. Therefore, the enrichment of lipid pathways in asthma is consistent with biological evidence. Regarding heptadecanoic acid [24], this odd chain saturated fatty acid (C17) is a constituent of dairy products [131]. An inverse association has been reported between odd chain saturated fatty acids and inflammation, along with oxidative stress [132]. 

As for the presence of 3-hydroxy tetradecanedioic acid in neonates who developed asthma during the first six years of life [20], this hydroxy fatty acid could be related to mitochondrial trifunctional protein deficiency [133], pantothenic acid deficiency or the inhibition of acetyl-CoA-requiring reactions due to stress (such as infection or asthma) [134]. Similarly, hydroxy fatty acids 2-hydroxyisobutyrate, 3-hydroxybutyrate, and 3-methyladipate [27] are involved in glucose and lipid metabolism [135]. Sinha et al. measured low levels of hydroxybutyrate in EBC of asthmatic children than in controls [103]. In the case of 3-hydroxybutyric acid, this molecule is synthesized in the liver from acetyl-CoA and is a ketone body that originates from fatty acids and ketogenic amino acids, leucine and isoleucine [44]. Accumulation of urinary hydroxy acids indicating a state of ketosis might reflect dysregulation of carbohydrate and amino acid metabolism along with energy depletion. 

The detection of phosphatidylglycerol, N-acryloylglycine and tiglylglycine in early-onset asthma [21] are associated with fatty acid metabolism, which is consistent with data suggesting lipid dysregulation in asthma [120,123]. Glycerophospholipid metabolism has been shown to be altered in BALF from animal models of allergic asthma [136] and in asthmatic subjects indicating the possible role of glycerophospholipid in the pathogenesis of asthma and of airway epithelial injury [50,137]. Phosphatidylglycerol is localized in mitochondria and its membranes and is a precursor for the synthesis of cardiolipin [138]. In mitochondria, cardiolipin maintains the inner mitochondrial membrane potential and supports proteins involved in mitochondrial respiration [138]. Therefore, it is possible that increased levels of phosphatidylglycerol, *N*-acryloylglycine and tiglyglycine in early-onset asthma mirrors the elevations in mitochondrial respiration required to support the increased work of breathing. 

Concerning the mild-moderate asthma phenotype [29], sphingolipid metabolism was enriched, which has been linked to bronchoconstriction and AHR in childhood asthma [139] and is consistent with adult studies [121].

### 3.6. Oxidative Stress

Oxidative stress has been acknowledged to be involved in the modulation of asthma inflammation [140]. Activated inflammatory cells in airways produce reactive oxygen and nitrogen species that contribute to asthma development and airway remodeling by reducing the ability of the airway epithelium to repair the damage [140]. An imbalance between oxidation and reducing systems was apparent by the recovery of serine, glycine and cysteine in asthmatic children [15,24,29,31,32,34], which are indicative of glutathione synthesis [101]. Likewise, in neonates [19], the presence of high indoxyl sulfate concentrations, a pro-oxidant metabolite [141]; whereas low 3, 4-dihydroxyphenyllactic acid methyl ester, ferulic acid 4-O-glucuronide [142] and 4-hydroxystachydrine (a urinary marker of citrus fruit consumption) [143], antioxidant molecules, suggest that exposure to early oxidative stress and suboptimal antioxidant protection might have a pathogenic role in the development of wheezing. 

Considering asthma phenotypes, Mattarucchi et al. found that urocanic acid, an anti-inflammatory metabolite, was reduced in children with atopic asthma [16]. The same conclusion was derived from research involving adult mild asthma patients [144]. Cis-urocanic acid is produced in the skin during sun exposure and is an intermediate in the conversion of histidine to glutamic acid [145]. Given that urocanic acid is able to suppress the immune response [145], then reduced levels observed in atopic asthma highlight the importance of adequate sun exposure via outdoor play for asthmatic children. The possibility that daily sun exposure could assist in the resolution of airway inflammation is intriguing and remains to be elucidated in intervention studies. 

Carraro et al. found a compound chemically related to retinoic acid, a derivative of vitamin A, markedly changed in EBC samples from severe asthma patients as compared to the non-severe [30]. Prior studies have reported that retinoids inhibit Th1 and promote Th2 immune responses along with abnormal airway repair and remodeling in asthma [146,147]. In murine models, high dietary vitamin A levels increased asthma severity [148]. 

The underlying mechanism proposed is that all-trans-retinoic acid (ATRA) accumulation in the lungs promotes asthma inflammation via activation of COX-2 expression and subsequent induction of prostaglandin pathway (PGD2 synthesis), including the synthesis of the Th2 cytokines IL4, IL5 and IL13 while decreasing IFNγ and, TNFα expression and IL12 synthesis in activated T-cells [146]. On the other hand, low serum vitamin A levels have been measured in asthmatic children, most likely due to increased utilization of pro-vitamin A carotenoid, β-carotene, as an antioxidant in response to oxidative stress associated with airway inflammation in asthma [149]. Carraro et al. also noted that calcitriol, the active form of vitamin D, characterized the lungs of healthy controls from the non-severe asthma groups [30]. There is growing attention on the possible link between vitamin D deficiency and asthma development in children. Hypovitaminosis D is common in asthmatic children compared to healthy children [150] and is associated with increased hospitalization [151], exacerbations [151], lung function impairment [152], increased asthma severity [153] and poorly controlled asthma [153,154]. Gupta et al. demonstrated an inverse correlation between vitamin D levels, airway smooth muscle mass and lung function in children with severe asthma [155]. Recently we published that sufficient plasma vitamin D concentrations (25(OH) D ≥ 25 ng/mL) in mild asthmatic children improved ventilatory function in central and peripheral airways as reflected by spirometry measures FEV_1_/FVC and FEF _25–75%_ [156]. Vitamin D is an immunomodulator, and vitamin D receptors (VDR) are expressed in airway epithelia, smooth muscle cells, lung fibroblasts and cells related to the immune system (macrophages, dendritic cells, monocytes, and activated T and B cells [157]. More specifically, in airways, VDRs regulate the transcription of genes implicated in inflammation and in the immune response [157]. Vitamin D is able to suppress the production of proinflammatory cytokines interleukin-17 (IL-17), IL-13 and promote anti-inflammatory IL-10 and Th2 cell activation [157], hence preventing the onset of asthma development.

Metabolic derangements in glutathione-cysteine redox balance were also found in corticosteroid-resistant severe asthma [15,31]. Specifically, two pathways were differentiated between severe asthma and the mild-moderate group: the serine and glycine pathway and the other with N-acylethanlolamine/N-acyltransferase. As explained earlier, serine is a precursor for glycine and cysteine synthesis, which are critical components of the antioxidant glutathione [101]. Park et al. noticed low levels of γ-glutamylcysteine in corticosteroid resistant non-respondents than in respondents and high concentrations of cysteine-glycine, which mirror increased degradation and reduced synthesis [31,158]. In light of asthma pathogenesis, excessive free radicals reactive oxygen species (ROS) and reactive nitrogen species (RNS) cause oxidative stress, which is implicated in disease development [159]. ROS and RNS contribute to airway inflammation by causing the oxidation and nitration of proteins vital for the resolution of inflammation and via induction of pro-inflammatory mediators, macrophages, cytokines and eosinophils [159]. Furthermore, the airways of asthmatic individuals were shown to have higher levels of ROS and RNS, which was associated with worse asthma severity [160] and poor control [71]. 

Comparatively, high levels of N-acylethanolamine phospholipids observed in the corticosteroid resistant asthma group [15] represent increased lipid peroxidation and dysregulation in thiol redox balance in severe asthma [160,161]. During cellular injury, N-acylethanolamine is formed by the action of membrane-associated N-acyltransferases [162]. This metabolite is also implicated in cell signaling [163] and protection against oxidative stress [164]. Hence, this study showed that disturbances related to oxidative stress might be potential therapeutic targets for severe ICS-resistant pediatric patients.

In terms of oxidative stress and inflammation, overproduction of ROS leads to degradation of polyunsaturated fatty acids (lipid peroxidation) in cell membranes generating hydrocarbons or alkanes in airways and urine [165]. Four studies found high levels of VOCs [2, 4-dimethylpentane, 2,4-dimethylheptane, 2-undecenal, octane, 2-methylpentane, 2-methylhexane, (C_13_H_28_), carbon disulfide, butanoic acid, 3-(1-methylethyl)-benzene, C_15_H_26_, benzoic acid] in exhaled breathe of asthmatic children as compared to transient wheezers and healthy controls [22,23,33], whereas low levels of acetone, 2,2,4-trimethylheptane, 1-methyl-4-(1-methylethenyl) Cyclohexen, 2,3,6- trimethyloctane, biphenyl, 2-ethenylnaphtalene, 2,6,10-trimethyldodecane [22,23], 1-penten-2-on, p-xylene and (C_11_H_24_) [33]. These metabolites are classified as hydrocarbons and are in agreement with previous studies in pediatric [33,166] and adult asthma [167,168]. Of note, Loureiro et al. showed that metabolites related to lipid peroxidation predicted lung function (as evaluated by FEV_1_), disease severity, Fractional exhaled nitric oxide (FeNO) and blood eosinophils and serum Ig E in adult asthma patients [167]. A plausible explanation for the presence of VOCs in exhaled breathe of asthmatic children might be that these metabolites represent by-products of inflammation-driven oxidation of polyunsaturated fatty acids found in cell membranes, whereas low levels of VOCs might result from the oxidation of long-chain hydrocarbons attributed to increased oxidative stress and airway remodeling in asthma patients [33].

In comparison, Gahleitner et al. quantified a different pattern of VOCs in asthmatic children (1-methylsulfonyl-propane, ethylbenzene, 1,4-dichlorobenzene, 4-isopropenyl-l-methyl cyclohexene, 2-octenal, octadecyne, 1-isopropyl-3-methylbenzene and 1,7-dimethyl-naphthalene [36] which are related to environmental exposure, asthma medication, and diet mediated by the metabolism of foods and additives found in commercial products (flavorings) [169]. Exposure to 1, 4-dichlorobenzene and 4-isopropenyl-1-methyl cyclohexene (also known as limonene), which is a component of citrus oil found in the peels of citrus fruits [170], have been linked to deficits in lung function and asthma in adults [171,172] and children [173]. 

### 3.7. Bile Acids

Two studies that used data collected during perinatal life discriminated bile acids [p-cresol-glucuronide, indoxyl sulfate, 1, 3, 7, 12 tetrahydroxycholan-24-oic acid, chenodeoxycholic acid, 3α-hydroxy-7, 1,2,-dioxo-5β-cholan-24-oic acid, 5-hydroxyindolepyruvate, glycocholic acid, taurochenodeoxycholate-3-sulfate] in the amniotic fluid of neonates that developed wheeze at 12 months [19] and another in urine samples of neonates that developed asthma at six years [20]. Remarkably, increased levels of urinary bile acids glycolithocholate, glycocholenate and glycohyocholate, as well as decreased tauroursodeoxycholate were reported in infants with atopy and wheeze at age 1 year, reflected gut microbial dysbiosis during the first 100 days of life [174]. In adult patients, Comhair et al. observed that asthmatics with high FeNO had higher plasma levels of taurocholate and glycodeoxycholate [50]. This is in accordance with the literature linking alterations in bile acids to inflammatory disorders [175], implicating that aberrations in gut microbiota might contribute to asthma in childhood. Given that the amniotic fluid contains both fetal and maternal metabolites, it is feasible that biliary acids are by-products of maternal gut microbiota [176,177]. Mice models have demonstrated that bile acids tauroursodeoxycholic and chenodeoxycholic acids attenuated allergic airway inflammation by inhibiting Th2 cytokines [178,179]. Incidentally, the amino acid taurine is eliminated from the body via bile acids, and previous studies in vivo and in vitro have demonstrated that NO modulated bile acid metabolism and bile production [50], which could explain increased levels measured in plasma and urine of asthmatics. High taurine levels have been found in the plasma of adult asthma patients [50].

### 3.8. Gut Microbiota

Emerging evidence has documented interaction between gut microbiota, the respiratory and immune systems [180]. An imbalance in the microbiome has been reported to precede asthma in school-aged children [181]. Gastrointestinal dysbiosis in the early neonatal period was associated with asthma in the first years of life [182]. Analysis of stool samples of 1 week and 1-month-old infants revealed a high microbial diversity that was positively associated with a lower incidence of asthma at 7 years of age, and this was not observed in 12 months old infants [181]. Intriguingly, differences in airway microbiota between asthmatic and non-asthmatics showed that the airways of asthmatic patients had increased Th2-derived pro-inflammatory cytokines IL-4, IL-5, and IL-13, thus suggesting that microbial dysbiosis could contribute to asthma progression in genetically predisposed individuals [180]. 

Three pediatric studies annotated gut microbial products 3-hydroxyhippuric acid, p-cresol, indoleacetic acid and benzoic acid in the urine of transient wheezers; and 4-(4-deoxy-α-d-gluc-4-enuronosyl)-d-galacturonate, indole, hydroxyphenyllactic acid [21], dimethylamine, Trimethylamine N- oxide (TMAO) and *N*-phenylacetylglycine in early-onset asthma [26,35]. These metabolites are derived from the action of intestinal microbiota on specific dietary components [181,183]. As for dimethylamine [35], this molecule is synthesized from TMAO. Chiu et al. reported that increased levels of *N*-phenylacetylglycine was correlated with food allergen-specific IgE [26], which is consistent with the literature [183]. Furthermore, in infants with bronchiolitis-induced wheeze [32], changes in fatty acid metabolism as represented by isobutyrylglycine, *N*-butyrylglycine and *N*-acetylneuraminic acids in urine samples could be related to an imbalance in gut microbiota. In high-risk infants for asthma, reduced fecal acetate concentrations, a short-chain fatty acid was associated with gut microbial dysbiosis [174]. Notably, high *N*-acetylneuraminic acid in the intestinal mucosa promoted bacterial growth by serving as a source of nutrients [184]. Thus, suggesting that a disruption in gut microbiota could predispose to bronchiolitis in infants. In the context of lung function, we reported a correlation between urinary 4-hydroxyphenylacetate and spirometric parameters FEV_1_ and FVC in mild-asthmatic pediatric patients [28], which is a marker of bacterial overgrowth and dysbiosis [185]. Anaerobic bacteria possess enzymes that are able to hydrolyze, deaminate and oxidize amino acids from dietary protein to tyrosine and finally to 4-hydroxyphenylacetic acid. On this note, dysbiosis of intestinal microbiota might be a determinant discriminating which children will develop allergic diseases, and therefore be suitable targets for therapeutic interventions either by modulation of microbial species or enzymes required to produce metabolites. 

### 3.9. Steroid Hormone Biosynthesis

Metabolites related to steroids (corticosterone, 2-methoxyestrone 3-sulfate, glucoronidate) and hormone biosynthesis mediated by phenylalanine metabolism (α-*N*-phenylacetyl-l-glutamine) were recovered in neonates that developed wheeze at 1 year [19] and asthma at 6 years [20]. In reference to high levels of glucoronidate in neonates not developing asthma at 6 years [20], studies have reported high cortisone [186] in the urine of healthy children compared to asthmatics. In contradiction, low levels of glucoronidate observed in asthmatic children might be caused by suppression of the hypothalamic-pituitary axis as a result of ICS treatment [187]. Decreased levels of plasma cortisone concentrations, including steroids (dehydroisoandrosterone sulfate, epiandrosterone sulfate and androsterone sulfate), were observed in adult asthmatics taking ICS [50,121]. It is feasible that in infants developing wheeze or asthma, increased stress might elevate blood glucoronidate levels and consequently increase the risk of developing systemic inflammation and asthma-like symptoms [188]. Then again, in this study, ICS therapy may have masked these effects.

### 3.10. Xenobiotics

There are multiple factors that can trigger asthma exacerbations, including environmental exposures. Park et al. identified 3,6-dihydronicotinic acid and 1,2-dihydronaphthalene in the urine of corticosteroid-resistant children [31]. Interestingly, both molecules are constituents of cigarette smoke [189] which highlights the effect of external factors such as passive smoking on asthma development in children.

## 4. Methods

We conducted a systematic literature search of the PUBMED database up to 23rd February 2021 to identify studies investigating the application of metabolomics in pediatric asthma and quantify metabolites associated with asthma pathobiology. Studies included human interventions that reported on the application of metabolomics or metabolic profiling in children from 0–18 years old and outcomes of asthma symptoms, lung function or asthma phenotypes. Furthermore, novel to this review is data complied on wheezing and bronchiolitis in neonates. Studies focusing solely on adults as defined in articles (>18 years) along with experiments performed in animals were excluded. Grey literature was not considered since these studies can vary considerably and are often affected by the low standard of quality, review and production. No study design, language or time restrictions were applied to eligible reports. Bibliographies of relevant studies were checked to identify additional publications missed by the search. Two authors (M.P., E.S.) screened titles and abstracts retrieved by the search and identified studies that potentially met the inclusion criteria outlined above. Then, full texts of potentially eligible studies were retrieved and assessed for eligibility. Hence, twenty articles were included in the final review. The authors (M.P., E.S.) extracted data from eligible studies that included study design characteristics and population details such as age group, author-date of study, follow-up, asthma assessment tool, biological specimen, metabolomic profiling technique, metabolites isolated, annotated biochemical pathways and study conclusions.

## 5. Limitations/Strengths

This review compiled up-to-date data on the use of metabolomics in pediatric asthma ranging from neonates through to 16 years of age. Strong evidence was presented on the utility of metabolic profiles in the clinical setting, and plausible interpretations in relation to asthma pathobiology were given for a vast range of metabolites. The heterogeneous nature of childhood asthma, as well as the intricate diagnosis based on conventional pulmonary function tests, require the identification of asthma biomarkers and associated molecular pathways that would aid in the prediction of asthma exacerbations, the development of early-onset asthma in infants, the discrimination of asthma and wheeze due to viral infections, along with the different asthma phenotypes, especially in corticosteroid resistant severe patients. Therefore, clinical practice will be guided towards personalized approaches in the treatment of symptoms, which inevitably will improve asthma control, the quality of life and decrease healthcare costs. Furthermore, in light of nutrient deficiencies identified from metabolic pathways, the possibility of diet modification and nutrition intervention therapy as a viable option to improve overall respiratory health and the patient’s quality of life is encouraging. 

A limitation of the afore-mentioned studies was the small sample size resulting in limited statistical power, as well as variability among study designs, populations studied, age groups, definition of wheezing and asthma (parent-reported versus physician confirmed) and asthma severity. With respect to diversity in metabolites obtained from exhaled breath condensate, urine, serum and VOCs, this emphasizes the importance of standardized criteria and metabolite assays for biosamples. The type and amount of metabolites detected can be influenced by a variety of factors such as the timing of sampling, collection procedure and processing, the stability of the storage, extraction, sample dilution, type and number of analytical methods used and assay for metabolites [190]. Another drawback, only one study assessed metabolites from more than one type of biospecimen [26]. In this context, it is difficult to compare metabolites across all biospecimens. Furthermore, external factors such as BMI, diet, physical exercise and treatment are known to influence the metabolome, which could mask the effects of the disease. Alternatively, minute changes in metabolites may not be detectable by standard analytical techniques. 

An additional source of heterogeneity is the metabolomic profiling technique NMR versus LC-MS, targeted versus non-targeted and statistical test employed for the mapping of metabolites and adjustment for confounding factors [13]. Compared to the method of LC-MS, NMR has less sensitivity [13]. The advantage of using an untargeted metabolomic profiling approach is that it captures all metabolites in a biological system as opposed to targeting specific metabolites, which may lead to the exclusion of important molecules associated with asthma progression and hinders replication and validation of findings across studies [13]. However, targeted metabolomics can accurately provide quantitative analysis of the metabolites of interest facilitating biomarker validation. Thus, a two-step approach employing untargeted metabolomics for biomarker discovery followed by targeted analysis will enable the validation and clinical application of metabolic biomarkers for the prediction and early diagnosis of asthma. Also, large longitudinal studies combining the metabolic changes in response to pharmacotherapy or related to disease progression will generate biomarkers for prognosis and treatment efficacy [191]. 

Regarding gaps in the literature and topics for further investigation, most studies focused on discriminating asthma from healthy pediatric patients [24,26,27,33,36]. There is an urgent need to study predictive biomarkers of asthma exacerbations, including discrimination of different asthma phenotypes that will assist in tailoring personalized medical therapy and better patient symptom care. To our knowledge, there is little data on the application of metabolomics in pediatric overweight asthma patients. One study has been performed in obese-asthma adult patients documenting a discrete respiratory, metabolic profile that deviates from obese or asthma patients alone [192]. In a study of children with obese-asthma phenotype, we found that overweight/obese children had lower FeNO measurements and supranormal spirometric parameters than normal-weight [193]. As described by Forno et al., there is a possibility that this abnormality is due to dysanapsis or incongruence in the growth of airways and lung parenchyma [194]. With respect to low FeNO values in overweight/obese asthmatic children, increased adiposity could lead to neutrophilic airway inflammation rather than eosinophilic, and FeNO is a surrogate marker for eosinophilic airway inflammation [195]. These findings have important implications in clinical practice because current asthma pharmacotherapy is based on treating eosinophilic airway inflammation and not for neutrophilic. As a result, obese-asthma in pediatric cases warrants for alternatives in therapeutic management.

In terms of the most optimal biospecimen, urine specimens could be a useful tool in predicting wheezing infants most likely to develop prior asthma manifestation of symptoms [27,32]. Apart from urine, VOC collection from exhaled breath is an attractive, non-invasive method approach that is suitable and acceptable for both infants and children [22,36]. The metabolomic approach could be a non-invasive, low-cost technique for discovering candidate markers that stratify patients by asthma phenotypes and plausible therapeutic targets. In the clinical setting, these studies will pave the way for introducing metabolic biomarkers in the care of pediatric asthma patients.

## 6. Conclusions

Asthma is one of the most common heterogeneous respiratory disorders in children. This condition, defined as a chronic inflammatory disease of the airways, is characterized by a variety of cellular and molecular mediators resulting in a wide spectrum of metabolic products. Metabolomics, the study of low-molecular-weight metabolites, enabled the discovery of metabolic signatures specific to pediatric asthma patients and sub-types, including potential pathogenic mechanisms. Alterations in metabolic pathways were associated with asthma pathology. The pathways and metabolites highlighted in this study will serve as a critical foundation for future studies to characterize the molecular structures and role in asthma pathogenesis, including clinical utility. 

## Figures and Tables

**Table 1:** Characteristics of pediatric asthma metabolomic studies reviewed from birth to school years.

Author Year/Study Design/ Country	Follow-Up	Population (*n*)	Group Allocated	Asthma or Bronchiolitis Diagnosis	Sample/ Metabolomic Technique	Metabolites Isolated	Annotated Pathways	Conclusions
	**NEONATES**							
Carraro et al., 2018 [19] Birth Cohort Netherlands	1 year	292 mothers @ 38–42 weeks gestation 142 analyzed.	wheezers *n* = 86 non-wheezers *n* = 56	Parent’s symptom report	Amniotic fluid (LC-MS) Untargeted	**High levels in the wheezing group:** Indoxyl sulfate, p-cresol glucuronide, 2-methoxyestrone-3-sulfate, S-adenosylhomocysteine, 1,3,7,12-tetrahydroxycholan- 24-oic acid, glycocholic acid, α- Ν-phenylacetyl-l-glutamine, corticosterone **High levels in non-wheezers:** 3-hydroxyphenylacetic acid, 3,4-dihydroxyphenyllactic acid methyl ester, ferulic acid 4-O-glucuronide, chenodeoxycholic acid 3- sulfate, 3a-hydroxy-7,12-dioxo-5β- cholan-24-oic acid, 4-hydroxystachydrine, 5-hydroxyindolepyruvate, dehydroepiandrosterone sulfate	Steroid hormone Biosynthesis, Phenylalanine metabolism, gluconeogenesis, bile acid synthesis, products of gut microbiota, oxidative stress and epigenetic dysregulation	Amniotic fluid collected at delivery differed in neonates that experienced wheezing at 1 year than in non-wheezers.
Chawes et al., 2019 [20] Birth Cohort Denmark	6 years	Asthmatic mothers COPSA C2000 *n* = 171 neonates @ 4 weeks of age. COPSAC 2010 *n* = 161	Persistent wheezers or asthmatics in the first 6 years of life	Physician	Urine (LC-MS) Untargeted	**Higher in asthmatic children vs. healthy controls:** Taurochenodeoxycholate-3-sulfate, 3-hydroxy-tetradecanedioic acid **Lower in asthmatic children vs. control:** Glucoronidated steroid	Steroid, fatty acid metabolism and bile acids	Metabolic profiles discriminated children developing asthma from healthy children. In both cohorts, urine metabolite levels measured at four weeks were related to asthma development before six years of age.
	**INFANTS**							
Chiu et al., 2018 [35] Longitudinal Taiwan	1, 2, 3, 4 years	PATCH Cohort *n* = 60	Asthma *n* = 30, Healthy controls *n* = 30	Physician	Urine (NMR) Untargeted	**Lower levels in asthmatic children vs. healthy controls:** Dimethylamine, allantoin, guanidoacetic acid, 1-methylnicotinamide	Purine and amino acid metabolism, nicotinamide/ nicotinate metabolism, methane metabolism and gut microbiota imbalance.	Metabolomic profiling provided a link of microbe-environment Interactions in the development of childhood.
Barlotta et al., 2019 [32] Prospective Italy	6 months, 12 months, 2 years	*n* = 52 @ 1 year old patients with acute bronchiolitis	Wheezers Non-wheezers	BronchiolitisPhysician	Urine (LC-MS) Untargeted	**Bronchiolitis-induced recurrent wheeze vs. non-wheezers:** Isocitrate, citric acid, oxoglutaric acid, lysine, cysteine, methionine. Isobutyrylglycine, *N*-butyrylglycine.	Citric acid cycle, fatty acid and amino acid metabolism, gut microbial dysbiosis	Metabolomic profiling of urine specimens from infants with bronchiolitis identified children at increased risk of developing recurrent wheezing.
Atzei et al., 2011 [34] Case-study Italy	N/A	*n* = 2 @ 33-37 weeks gestation < 28 days Patients with RSV bronchiolitis		Physician	Urine (NMR)	Associated with RSV bronchiolitis: Betaine, creatinine, glycine	Creatine metabolism and epigenetic regulation	1H-NMR can be potentially applied to identify metabolic alterations in urine samples related to the differences in the inflammation of bronchioles.
Turi et al., 2018 [25] INSPIRE Cohort USA	1, 2, 3 years	*n* = 140 120 days old	Healthy *n* = 60, HRV *n* = 10, RSV *n* = 70	Physician	Urine (NMR) Untargeted	11 metabolites were significantly different between **RSV ARI, HRV ARI vs. healthy control infant groups**: 1-methylnicotinamide, 4-deoxythreonic acid, citrate, creatine, hypoxanthine, alanine, succinate, 3-hydroxyisovalerate, acetone, valine, 2-aminobutyrate	Citric acid cycle, amino acid metabolism, nicotinamide/ nicotinate metabolism, catecholamine biosynthesis, glucose-alanine cycle, glutamate metabolism, arginine-proline metabolism	Metabolomics may aid in prophylaxis against bronchiolitis in infants.
**PRE-SCHOOL**								
Carraro et al., 2018 [21] Cohort Italy	3 years	*n* = 47 2–5 years	Wheezing *n* = 34, Healthy *n* = 13	Physician	Urine (LC-MS) Untargeted	**Higher levels in transient wheezers vs. early-onset asthma:** Oxoadipic acid, epinephrine, L-tyrosine, 3-hydroxyhippuric acid, benzoic acid, 3 hydroxy-sebacic acid, dihydroferulic acid 4-sulfate, *p*-cresol, indolelactic acid, *N*-acetyl-l-phenylalanine, *N*2-acetyl-ornithine **Higher levels in early-onset asthma vs. transient wheezers:** 4-(4-deoxy-α-d-gluc-4-enuronosyl)-d-galacturonate, glutaric acid, 4-hydroxy nonenal, phosphatidylglycerol, 3-methyluridine, steroid O-sulfate, 5-hydroxy-l-tryptophan,3-indoleacetic-acid, tiglylglycine, indole, cytosine, *N*-acetylputrescine, indole-3-acetamide, 6-methyladenine, 5-methylcytosine, *N*-acryloylglycine, hydroxyphenyllactic acid.	Tryptophan metabolism, fatty acid metabolism and microbial derivatives	Urine metabolites distinguished between transient wheezers and early-onset asthma.
Smolinska et al., 2014 [22] Prospective Cohort Netherlands	6 years	ADEM Study *n* = 252 2–4 years	Recurrent Wheeze *n* = 202 Healthy controls *n* = 50 At age 6 years: Healthy *n* = 49, Transient wheezers *n* = 121, Early-onset Asthma *n* = 76	Physician	VOC (GC-MS) Targeted	High levels in early-onset asthma vs. transient wheezers: 2,4-dimethylpentane, 2,4-dimethylheptane, 2-undecenal, octane, 2-methylpentane, 2,4-demethylheptane, 2-methylhexane Low levels in early-onset asthma vs. transient wheezers: Acetone, 2,2,4-trimethylheptane, 1-methyl-4-(1-methylethenyl) Cyclohexen, 2, 3, 6-trimethyloctane, biphenyl, 2-ethenylnaptalene, 2, 6, 10-trimethyldodecane	Hydrocarbons produced during lipid peroxidation	VOCs profile in exhaled breath discriminated healthy, transient wheezing and true asthmatic children. VOCs predictive of early-onset asthma.
Klaassen et al., 2015 [23] Prospective Cohort Netherlands	6 years	ADEM study *n* = 202 2–4 years	Recurrent wheezers n= 202 At age 6 years: Healthy n = 4, Asthma n =76, Transient wheeze n =122	Physician	VOC (GC-TOF-MS) Targeted	**High levels in asthmatics:** Octane, 2-methylhexane, 2, 3, 6 -trimethyloctane, 2, 4-dimethylheptane **Low levels in asthmatics:** Acetone, 2-undecenal, 2, 6, 10-trimethyldodecane, 2,4-dimethylpentane, 2-methylpentane	Hydrocarbons produced during airway inflammation	VOCs profile plus Asthma Predictive Index (API) status improved asthma diagnosis at preschool age. VOCs could be a valuable monitoring tool for airway inflammation and in predicting asthma onset.
Chiu et al., 2020 [26] Cross-sectional Taiwan	N/A	*n* = 54 3–5 years	Asthma *n* = 28, Control *n* = 26	Physician	Plasma Urine (NMR) Untargeted	**Higher in asthma vs. control:** Histidine **Lower in asthma vs. control:** 1-methylnicotinamide, trimethylamine N-oxide (TMAO). **Related to allergic sensitization (Ig E)/Food allergy:** *N*-phenylacetylglycine, pyruvate, valine, leucine, isoleucine	Histadine metabolism, nicotinamide and pyruvate metabolism, phenylalanine metabolism, amino acid metabolism and products of microbial metabolism	Plasma pyruvate metabolism associated with Ig E production. Urinary branched-chain amino acids were associated with food allergic reactions.
	**SCHOOL CHILDREN**							
Saude et al., 2011 [27] Cross-sectional Canada	N/A	*n* = 135 4–16 years SAGE Birth Cohort	Stable *n* = 73, Unstable asthma *n* = 20, Healthy controls *n* = 42	Physician	Urine (NMR) Targeted	**Protective against asthma** **exacerbation:** 1-methylnicotinamide **Asthma vs. healthy controls:** 1–methylhistamine, 1-methyl-nicotinamide, 2- methylglutarate, 2-oxoglutarate, 3-OH-3-methyl-glutarate, 3-methyladipate, 4-aminohippurate, acetone, adenine, alanine, creatine, dimethylamine, formate, fumarate, glucose, glycolate, imidazole, lactate, methylamine, O-acetylcarnitine, oxaloacetate, phenylacetylglycine, phenylalanine, tryptophan, tyrosine, cis-aconitate, Myo-inositol, trans-aconitate. **Separating stable vs. unstable** asthma: 2-oxaloglutarate, succinate, fumarate, 3-hydroxy 3-methylglutarate, threonine, aconitate, acetylcarnitine, trimethylamine, threonine, taurine, 4-aminohippurate **Stable vs. unstable vs. healthy controls**: 4-aminohippurate, carnitine, homovanillate, kynurenine, O-acetylcarnitine, succinate, taurine, threonine, trimethylamine.	Citric acid cycle, nicotinamide metabolism, lipid metabolism, Protein metabolism, purine metabolism, glucose metabolism, tryptophan metabolism, histamine biosynthesis including catecholamine synthesis	^1^H-NMR can be used to differentiate stable asthma from controls and unstable asthma.
Tao et al., 2019 [24] Cohort China	N/A	*n* = 109 6–11 years	Healthy *n* = 29, Uncontrolled asthma *n* = 37, Controlled asthma *n* = 43	Physician	Urine (GC-MS) Untargeted	**Asthma diagnosis and discrimination of controlled vs. uncontrolled asthma:** Uric acid, stearic acid, threitol, acetylgalactosamine, heptadecanoic acid, aspartic acid, xanthosine, hypoxanthine **Healthy vs. uncontrolled/** **Controlled asthma:** Glycine, serine, threonine, Pantothenate and CoA synthesis, BCAA synthesis, tyrosine, inosine, adenosine, arginine, proline, alanine, aspartate, glutamate, pyruvate, tryptophan	Citric acid cycle, purine metabolism, lipid and carbohydrate metabolism, amino acid and phenylalanine metabolism, pantothenate and Coenzyme A biosynthesis	Urine metabolomics discriminated asthma as well as controlled and uncontrolled sub-types and elucidated the biological mechanisms of pediatric asthma.
Dallinga et al., 2010 [33] Prospective Netherlands	N/A	*n* = 120 5–16 years	Asthma *n* = 63, Healthy controls *n* = 57	Physician	VOC (GC-TOF-MS) Untargeted	**Metabolites differentiated between asthma vs. healthy controls:** Branched hydrocarbons (C_13_H_28_, C_11_H_24_), carbon disulfide, 1-penten-2-on, butanoic acid, 3-(1-methylethyl)-benzene, unsaturated hydrocarbon (C_15_H_26_), benzoic acid, *p*-xylene	Hydrocarbons produced during lipid peroxidation	EBC samples and comparing VOCs differentiated children with asthma from healthy controls.
Gahleitner et al., 2013 [36] Experimental UK	N/A	*n* = 23 8–16 years	Asthma *n* = 11, Healthy *n* = 12	Physician	VOC (GC-MS) Targeted	**Metabolites differentiated between asthma vs healthy:** 1-(methylsulfanyl)propane, ethylbenzene, 1,4-dichlorobenzene, 4-isopropenyl-1-methylcyclohexene, 2-octenal, octadecyne, 1-isopropyl-3-methylbenzene, 1,7 dimethylnaphtalene	Organic compounds from external sources. Used in food manufacturing (flavorings) and disinfectants.	VOCs discriminated between asthmatic and healthy children. The application of breath markers could be a potential non-invasive and low-cost technique for the management of pediatric asthma.

Key: @: at; vs.: versus; VOCs: Volatile Organic Compounds; GC-MS: Gas-Chromatography-Mass Spectrometry; GC-TOF-MS: Gas-Chromatography-time of flight-Mass Spectrometry; LC-MS: Liquid Chromatography-Mass Spectrometry; NMR: ^1^H Nuclear Magnetic Resonance; EBC: Exhaled Breath Condensate; RSV: Respiratory Syncytial Virus; HRV: Human Rhino Virus; ARI: Acute Respiratory Infection; N/A: Not applicable; BCAA: Branched-Chain Amino Acids; Ig E: Immunoglobulin E.

**Table 2:** Characteristics of metabolomic studies reviewed on pediatric asthma phenotypes.

Asthma Phenotype	Author Year/ Study Design /Country	Population (*n*)	Group Allocated	Asthma Diagnosis	Sample/ Metabolomic Technique	Metabolites Isolated	Annotated Pathways	Conclusions
**Mild**	Papamichael et al., 2019 [28] Cross-sectional Greece	*n* = 65 5–12 years	N/A	Physician ACQ	Urine (GC-MS) Targeted	**Metabolites correlated with PFTs (FEV_1_, FVC, FEV_1_/FVC, PEF, FeNO) and asthma control:** Lactic, 4-hydroxyphenylacetic, 5-hydroxyindoleacetic, glycolic, malic acid.	Tryptophan and tyrosine metabolism, lactic acidosis, catecholamine synthesis and alterations in gut microbiota.	Metabolomics is a promising approach in the research for novel biomarkers for asthma monitoring
**Mild-Moderate**	Kelly et al., 2017 [29] Cohort Costa Rica	*n* = 380 6–14 years	N/A	Physician	Plasma (LC-MS) Untargeted	574 Metabolites isolated in mild-moderate asthmatics. 91 associated with AHR, 102 with pre- FEV_1_/FVC and 155 with post-FEV1/FVC 24 metabolites common to all 3 parameters	Metabolites common to AHR, pre and post-FEV_1_/FVC related to: Glycerophospholipids, linoleic acid and pyrimidine metabolism. **Metabolites pertaining to AHR:** Disturbances in Glycerophospholipid and linoleic acid metabolism, d -glutamine/ glutamate, sphingolipid and pyrimidine metabolism, as well as Nitrogen metabolism. **Pre and post bronchodilation FEV_1_/FVC:** Citric acid cycle, lipid metabolism, alanine, aspartate and glutamate metabolism, arginine—proline metabolism, glycine, threonine and serine metabolism, pyrimidine metabolism, BCAA and nitrogen metabolism, pantothenate and CoA biosynthesis and aminoacyl tRNA biosynthesis. **Post FEV_1_/FVC:** Pantothenate and CoA biosynthesis	Metabolites and metabolomic profiles distinguished children with asthma by the degree of lung function as reflected by spirometric parameters, thus confirming the existence of an asthma severity metabolome.
**Severe**	Carraro et al., 2013 [30] Cross-sectional Italy	*n* = 57 8–17 years	Severe *n* = 11, Non-severe *n* = 31 (17 taking medication) Healthy controls *n* = 15	Physician	EBC (LC-MS) Untargeted	**Severe asthma:** Retinoic acid, deoxyadenosine **Non-severe:** 20-hydroxy-PGF2a, Thromboxane B2, 6 keto-prostaglandin F1a **Healthy controls:** Ercalcitriol (active vitamin D2)	Compounds related to: Retinoic acid, adenosine and vitamin D	Breathomics discriminated between severe, non-severe and healthy child asthmatics.
**Cortico-** **steroid** **Resistant**	Fitzpatrick et al., 2014 [15] Cross-sectional US	*n* = 57 6–17 years	Mild asthma *n* = 22, Severe *n* = 35	Physician	Plasma (LC-MS) Untargeted	**Severe asthma:** Glycine, serine, threonine, *N*-acylethanolamine, *N*-acyltransferase pathway	Biosynthesis of purine /pyrimidines, phospho-glycerides, sphingo-lipid, glycolipids. Folate cycle, glutathione synthesis. Oxidative stress	Metabolomics revealed that oxidative stress is a contributory factor to corticosteroid refractory severe asthma in children.
**Cortico-** **steroid** **Resistant**	Park et al., 2017 [31] Cross-sectional US	*n* = 30 6–17 years	Corticosteroid resistant *n* = 15, Corticosteroid responders *n* = 15	Physician	Urine (LC-MS) Untargeted	Metabolites discriminating **corticosteroid responders from non-responders**: 3,6-dihydronicotinic, 3-methoxy-4-hydroxyphenyl(ethylene)glycol, 3,4-dihydroxy-phenylalanine, γ-glutamylcysteine, Cys-Gly, reduced Flavin mononucleotide **High in the corticosteroid resistant (non-responders) group:** Cyst-Gly 3,6-dihydronicotinic, 3,4-dihydroxy-phenylalanine, 1,2-dihydronaphthalene- 1,2-diol, 3-methoxy-4-hydroxyphenyl(ethylene)glycol **Low in corticosteroid resistant group (non-responders)**: γ-glutamylcysteine	Tyrosine metabolism, catecholamine biosynthesis, and glutathione metabolism. 3,6-dihydronicotinic and 1,2-dihydronaphthalene- 1,2-diol are present in cigarette smoke	Putative biomarkers isolated using the metabolomics approach differentiated corticosteroid resistant non-responders) from responders in pediatric asthma.
**Atopic**	Mattarucchi et al., 2012 [16] Cross-sectional Italy	Atopic *n* = 41, Healthy *n*= 12 Median age 11 years	Well-controlled with β-agonists *n* = 14, Well-controlled with daily controller drugs *n* = 16, Poorly-controlled with daily controller drugs *n* = 11	Physician	Urine (LC-MS) Untargeted	**Low levels in asthmatics**: Urocanic, methyl-imidazoleacetic, Ile-Pro fragment	Histamine metabolism. Urocanic acid related to inflammation/immunity and Ile-Pro to prolidase activity.	Metabolic profiling offers the potential of asthma characterization and identification of inflammation- related metabolites.

Key: GC-MS: Gas-Chromatography-Mass Spectrometry; LC-MS: Liquid Chromatography-Mass Spectrometry; EBC: Exhaled Breath Condensate; PFTs: Pulmonary function tests; AHR: Airway Hyperresponsiveness; Ile-Pro: Isoleucyl-Proline: Cys-Gly: Cysteine-Glycine; ACQ: Asthma Control Questionnaire; N/A: Not Applicable; BCAA: Branched-Chain Amino Acids; Co A: Coenzyme A; FEV_1_: Forced Expiratory Volume in 1 second; FVC: Forced Vital Capacity; FEV_1_/FVC: Ratio of Forced Expiratory Volume in 1 second and Forced Vital Capacity; PEF: Peak Expiratory Flow; FEF _25–75%_: Forced Expiratory Flow at 25–75% of the pulmonary volume; FeNO: Fractional exhaled Nitric Oxide.
